# Prevalence of Clinical Manifestations Known to Be Associated With Insulin Resistance Among Female Medical Students of a Private College in Saudi Arabia

**DOI:** 10.7759/cureus.72905

**Published:** 2024-11-02

**Authors:** Yousria Badawy, Rana B Atef, Sarah Faour, Yara Jastania, Raneem Alathath, Farah Alkhotany

**Affiliations:** 1 Family Medicine, Ibn Sina National College for Medical Studies, Jeddah, SAU; 2 Family and Community Medicine, Ibn Sina National College for Medical Studies, Jeddah, SAU

**Keywords:** acne, carb cravings, day sleep, hidradenitis, hirsutism, memory loss, no concertation, psoriasis, s: insulin resistance, skin manefestation suppurativa

## Abstract

Background

Insulin resistance has a significant relationship with a lot of chronic diseases. This is an alarm for the future high prevalence of many chronic diseases. Due to the lack of sufficient data surrounding the clinical manifestations of insulin resistance among the Saudi population, the study aims to fill this gap by assessing the prevalence of dermatological, cognitive, daytime sleep, and craving changes in the Saudi population, which are markers of insulin resistance.

Methods

A cross-sectional study was carried out to study the prevalence of clinical manifestations known to be associated with insulin resistance with a convenient nonprobability sample among 272 female medical students at the Ibn Sina National College for Medical Studies, a private medical college in Jeddah, Saudi Arabia. Each participant underwent an in-person interview where height, weight, and waist circumference were measured and BMI calculated. The in-person interview was performed using a series of questionnaires that assessed demographic data and clinical manifestations related to insulin resistance. Data was collected and grouped using Microsoft Excel (Microsoft Corp., Redmond, WA, USA). Statistical analyses were performed using IBM SPSS Statistics for Windows, version 22 (IBM Corp., Armonk, NY, USA). For prevalence calculations, percentages were used. The Chi-square test was used to analyze qualitative variables. The level of significance adopted was p < 0.05.

Results

The research revealed that acanthosis nigricans, skin tags, and alopecia areata were statistically significant in relation to waist circumference as an indicator of insulin resistance. Conversely, of all the dermatological manifestations, acne, psoriasis, hidradenitis suppurativa, hirsutism, and vitiligo were not associated with waist circumference. Also, polycystic ovary syndrome (PCOS) was statistically significant. Regards cognitive changes, carbohydrate craving, and day sleep were all proportionally higher in the high waist circumference category but were not found to be significantly significant in relation to waist circumference.

Conclusion

The findings of the study confirm the association of dermatological and endocrine features such as acanthosis nigricans, skin tags, alopecia areata, and PCOS with waist circumference as an indicator of insulin resistance. The high prevalence of increased waist circumference in the study population is also in line with international data concerning the increasing incidence of insulin resistance and, consequently, diabetes mellitus will be seen among younger age groups.

## Introduction

Insulin resistance is defined clinically as an individual's inability to increase glucose uptake and utilization as much as a normal individual with an equivalent amount of exogenous or endogenous insulin [1].

Dermatological manifestations of insulin resistance are well-reported. These include acanthosis nigricans, skin tags, hirsutism, hidradenitis suppurativa, vitiligo, and alopecia. Activation of fibroblast and keratinocyte insulin growth factor 1 (IGF-1) is thought to be the mechanism behind these changes, as well as insulin’s role in sex steroid synthesis [2].

Excessive daytime sleepiness (EDS) has been demonstrated in patients suffering from metabolic syndrome, which is known to result from resistance to insulin. A study demonstrated an Epworth Sleepiness Scale (ESS) of 8.263 ± 5.7 in metabolic syndrome subjects, a figure that is 1.8 times higher than that seen in the general population [3].

Brain activity changes in reward-processing regions are correlated with insulin and glucose levels. Food cues activate limbic-striatal regions in the setting of mild blood glucose declines, and hypothalamic-striatal connectivity is higher with increased glucose levels. Carbohydrate cravings are therefore a behavioral response to addiction-like brain changes [4].

There is very little published data concerning the prevalence of insulin resistance and, more specifically, its manifestations among the Saudi population. A study conducted among a population of 638 females residing in Alkharj, Saudi Arabia, found an 18.8% prevalence of prediabetes, and a prevalence of 3.8% for diabetes, as determined by HbA1C (glycated hemoglobin) levels [5]. There is scant evidence that assesses the prevalence of the clinical manifestations of insulin resistance in the Saudi population. A study conducted among dermatology clinic attendants found an increased incidence of acanthosis nigricans among obese participants, and increased skin tag incidence among obese males, but not obese females [6]. Also, they mentioned that the incidence values, however, cannot be extrapolated to the Saudi general population as they were recruited from dermatology clinics. Another study found relatively high incidences of hirsutism (47.3%), acne vulgaris (40.6%), and androgenic alopecia (20.3%) among Saudi females with polycystic ovaries but as with the previous study, this prevalence cannot be generalized to the rest of the population [7]. As for excessive daytime sleepiness, one study conducted among students of Taif University found a prevalence of 15.29% using the ESS, with significant correlations between EDS and body weight, body mass index, waist-to-hip ratio, fasting and postprandial blood glucose, fasting serum insulin and HOMA-IR (Homeostatic Model Assessment for Insulin Resistance) index [8]. Due to the lack of sufficient data surrounding the clinical manifestations of insulin resistance among the Saudi population, the study aims to fill this gap by assessing the prevalence of dermatological, cognitive, daytime sleep, and craving changes in the Saudi population, which are markers of insulin resistance. The study was conducted in a private medical college, as medical students are exposed to at least one or more of the risk factors of insulin resistance due to their stressful lifestyles, which in turn is known to detrimentally affect health and diet.

## Materials and methods

Study design and setting

A cross-sectional study was carried out to study the prevalence of clinical manifestations known to be associated with insulin resistance among female medical students at the Ibn Sina National College for Medical Studies, a private medical college in Jeddah, Saudi Arabia.

The study population consisted of female medical students attending the medicine program of all academic year levels (from year one to year six). The period of data collection was from March to April 2022. Among the inclusion criteria for participation in the study was enrollment in a private college. Exclusion criteria were pregnancy and/or lactation and failure to complete the study measurements.

Sampling technique

A convenient nonprobability sampling technique was used. The sample size was determined based on a single population formula using Epi-info version 7 (Centers for Disease Control and Prevention {CDC}, Atlanta, GA) and the calculated sample size was 214, based on an estimated prevalence of 23% as established from a previous study in a similar cohort with a margin of error of 5% [9].

Data collection

Each participant underwent an in-person interview and had anthropometric measurements taken. The in-person interview was performed using a series of questionnaires that assessed demographic data and clinical manifestations related to insulin resistance. For assessing clinical manifestations, data concerning dermatological changes, cognitive affection, carbohydrate cravings, and the presence or absence of day sleep were assessed. For day sleep, the ESS was used [10]. BMI was calculated using weight and height. The cut-off point for waist circumference was less than 88 cm which was considered normal but 88 or more was considered high waist circumference.

Validity and reliability

The ESS for daytime sleepiness has been shown to have good internal consistency and construct validity; however, one study did show that participants tended to underestimate daytime sleepiness [11].

The tools used for assessing cognitive changes, dermatological manifestations, and carbohydrate cravings were designed by the researchers, due to a lack of suitable validated tools. However, the questions were simple and straightforward, and in the case of the dermatological manifestation questionnaire, images of the relevant cutaneous signs were used in an attempt to facilitate participant understanding and increase reliability.

Statistical analysis

Data was collected and grouped using Microsoft Excel (Microsoft Corp., Redmond, WA, USA). Statistical analyses were performed using IBM SPSS Statistics for Windows, version 22 (IBM Corp., Armonk, NY, USA). For prevalence calculations, percentages were used. The Chi-square test was used to analyze qualitative variables. The level of significance adopted was p < 0.05.

Ethical consideration

Approval for this study was obtained from the Ibn Sina National College for Medical Studies, Jeddah. All information obtained was kept strictly confidential. The data collection sheet included a consent for participation. The approval number is IRRB-04-28022022.

## Results

Table 1 represents the sociodemographic data and clinical profile among female medical students in Ibn Sina National College for Medical Studies, a private medical college in Jeddah, Saudi Arabia. A total of 272 medical students participated in the study with a response rate of 100%. Among the respondents just above half (51.1%) were in the age range of 20-22 years and more than one-fourth (26.8%) were in the age group of 23-25. Students from all levels participated in the study but mainly were from the second year (23.2%) and fourth year (23.9%). A minority (12.4%) of the students in the sample were former or current smokers. As regards to the body mass index (BMI) category less than one-third (30.5%) were overweight or obese. Likewise, less than one-fourth (23.2%) of participants had a high waist circumference.

**Table 1:** Socio-demographic data and clinical profile among female medical students of a private medical college in Saudi Arabia, 2022 (n = 272).

Variable		Frequency	Percentage (%)
Age group (in years)	Less than 20	49	18.0
	20 - 22	139	51.1
	23 - 25	73	26.8
	25 - 28	10	3.7
	More than 28	1	0.4
Level of education	First	45	16.5
	Second	63	23.2
	Third	49	18.0
	Fourth	65	23.9
	Fifth	27	9.9
	Sixth	23	8.5
Smoking status	Never	237	87.1
	Former and current	35	12.9
BMI categories (kg/m^2^)	Normal	189	69.5
	Overweight or obese	83	30.5
Waist circumference (cm)	Normal waist	209	76.8
	High waist	63	23.2

The health status data of the participants are shown in Table 2. Among all the females who participated in the study, a small minority had dyslipidemia (2.6%) or hormonal disorders (4.8%). In addition to that a small fraction had a history of diabetes mellitus (0.4%) and hypertension (0.7%). At the same time, a minority (4%) of the sample admitted to the usage of corticosteroids.

**Table 2:** Health status data among female medical students at a private medical college in Saudi Arabia, 2022 (n = 272).

Variable		Frequency	Percentage (%)
Dyslipidemia	Yes	7	2.6
	No	265	97.4
Diabetes mellitus	Yes	1	0.4
	No	271	99.6
Hypertension	Yes	2	0.7
	No	270	99.3
Hormonal disorders	Yes	13	4.8
	No	259	95.2
Corticosteroids	Yes	11	4.0
	No	261	96.0
Total		272	100.0

The prevalence of dermatological and gynecological manifestations common with insulin resistance of female undergraduate medical students is presented in Table 3. Among all participants, more than one-third (36.4%) complained of having acne. Skin tags were present in 6.6% of all participants. Moreover, acanthosis nigricans were a complaint of 5.9% of the studied sample. On the other hand, a small percentage of the participants had hirsutism (3.7%) and alopecia areata (3.3%). In addition, a minority (2.9%) reported having psoriasis and hidradenitis suppurativa (2,2%). A minority (8.1%) revealed having vitiligo. Lastly, a tiny fraction (0.7%) of the sample reported a personal diagnosis of polycystic ovarian syndrome.

**Table 3:** Prevalence of dermatological and gynecological manifestations common with insulin resistance among female medical students of a private medical college in Saudi Arabia 2022 (n = 272). PCOS: polycystic ovary syndrome.

Variable		Frequency	Percentage (%)
Acne	Yes	99	36.4
	No	173	63.6
Acanthosis nigricans	Yes	16	5.9
	No	256	94.1
Skin tags	Yes	18	6.6
	No	254	93.4
Alopecia areata	Yes	9	3.3
	No	263	96.7
Psoriasis	Yes	8	2.9
	No	264	97.1
Hidradenitis suppurativa	Yes	6	2.2
	No	266	97.8
Hirsutism	Yes	10	3.7
	No	262	96.3
Vitiligo	Yes	22	8.1
	No	250	91.9
PCOS	Yes	2	0.7
	No	270	99.3

Clinical manifestations among the sample female group are revealed in Table 4. Overall, less than one-third (30.5%) of female medical students had foggy brains. Also, students who have difficulty concentrating accounted for another one-third (34.9%). Regarding struggling with planning and solving problems there were 32.7% of the total number of students. Those who have memory which became worse in the past few years were around 30% and those who have difficulty in understanding written or verbal information were only 14.3%. Those who had excessive day sleep were 23.9% of the tested population. Those who felt hungry even after eating some sweets were around one-fourth (25.7%), but those who usually had extreme thirst or hunger were around one-third (31.3%).

**Table 4:** Prevalence of some clinical manifestations known to be associated with insulin resistance among female medical students of a private medical college in Saudi Arabia, 2022 (n = 272).

Variable		Frequency	Percentage (%)
Sleep			
Day sleep	Unlikely day sleep	207	76.1
	Excessive day sleep	65	23.9
Cognitive changes			
Foggy brain	Yes	83	30.5
	No	189	69.5
Difficulty concentrating	Yes	95	34.9
	No	177	65.1
Struggling with planning and problem-solving	Yes	89	32.7
	No	183	67.3
Worsening memory	Yes	84	30.9
	No	188	69.1
Difficulty understanding	Yes	39	14.3
	No	233	85.7
Carbohydrate cravings			
Sweet cravings	Yes	98	36.0
	No	174	64.0
Hunger after eating	Yes	70	25.7
	No	202	74.3
Extreme thirst or hunger	Yes	85	31.3
	No	187	68.7

Table 5 shows that of all the dermatological manifestations, acne was reported as the highest in both groups (37.3% and 33.3% in the high and normal waist circumference groups, respectively), but did not demonstrate statistical significance with waist circumference. Similarly, psoriasis, hidradenitis suppurativa, hirsutism, and vitiligo were not associated with waist circumference. Conversely, acanthosis nigricans (p = 0.001), skin tags (p = 0.033), and alopecia areata (p = 0.006) were statistically significant in relation to waist circumference as an indicator of insulin resistance. A minority (4.3%) of participants belonging to the normal waist circumference category reported having a polycystic ovary, compared to 20.6% in the high waist circumference category. This association was found to be statistically significant (p = 0.001).

**Table 5:** Association of dermatological and gynecological manifestations with waist circumference as an indicator of insulin resistance among female medical students of a private college in Saudi Arabia, 2022 (n = 272). PCOS: polycystic ovary syndrome.

Variable	Waist circumference			
		Normal (n = 209)	High (n = 63)	p value
Acne	Yes	78 (37.3)	21 (33.3)	.564
	No	131 (62.7)	42 (66.7)	
Acanthosis nigricans	Yes	2 (1.0)	14 (22.2)	.001
	No	207 (99.0)	49 (77.8)	
Skin tags	Yes	10 (4.8)	8 (12.7)	.033
	No	199 (95.2)	55 (87.3)	
Alopecia areata	Yes	3 (1.4)	6 (9.5)	.006
	No	206 (98.6)	57 (90.5)	
Psoriasis	Yes	6 (2.9)	2 (3.2)	.589
	No	203 (97.1)	61 (96.8)	
Hidradenitis suppurativa	Yes	6 (2.9)	0 (0.0)	.202
	No	203 (97.1)	63 (100.0)	
Hirsutism	Yes	5 (2.4)	5 (7.9)	.055
	No	204 (97.6)	58 (92.1)	
Vitiligo	Yes	0 (0.0)	2 (3.2)	.053
	No	209 (100.0)	61 (96.8)	
PCOS	Yes	9 (4.3)	13 (20.6)	.001
	No	200 (95.7)	50 (79.4)	

Table 6 shows the relation between waist circumference as an indicator of insulin resistance and some clinical manifestations known to be associated with insulin resistance. Within the cognitive changes investigated, Table 6 shows that participants were asked if they experienced a foggy brain, difficulty in concentrating, struggles with planning and problem-solving, worsening memory, or difficulty in understanding, none of which were found to have a statistically significant correlation with waist circumference. However, all these variables were proportionately higher in the high waist circumference subgroup, except for difficulty understanding, which was calculated as 14.4% in the normal waist circumference subgroup, and 14.3% in the high waist circumference subgroup, a difference of 0.1%. Similarly, questions that pertained to carbohydrate cravings such as craving sweets after consuming something sweet, hunger after eating, and extreme thirst or hunger, were all proportionately higher in the high waist circumference category but were not found to be statistically significant in relation to waist circumference. As regards day sleep, determined by the ESS, it did not demonstrate a statistically significant relationship with waist circumference as an indicator of insulin resistance.

**Table 6:** Association of some clinical manifestations with waist circumference as an indicator of insulin resistance among female medical students of a private college in Saudi Arabia, 2022 (n = 272).

Variable	Waist circumference			
		Normal (n = 209)	High (n = 63)	p value
Cognitive changes				
Foggy brain	Yes	63 (30.1)	20 (31.7)	.809
	No	146 (69.9)	43 (68.3)	
Difficulty concentrating	Yes	67 (32.1)	28 (44.4)	.071
	No	142 (67.9)	35 (55.6)	
Struggles with planning problem-solving	Yes	63 (30.1)	26 (41.3)	.099
	No	146 (69.9)	37 (58.7)	
Worsening memory	Yes	59 (28.2)	25 (39.7)	.085
	No	150 (71.8)	38 (60.3)	
Difficulty understanding	Yes	30 (14.4)	9 (14.3)	.989
	No	179 (85.6)	54 (85.7)	
Carbohydrate cravings				
Sweet cravings	Yes	69 (23.0)	29 (46.0)	.059
	No	140 (77.0)	34 (54.0)	
Hunger after eating	Yes	50 (23.9)	20 (31.7)	.213
	No	159 (76.1)	43 (68.3)	
Extreme thirst or hunger	Yes	59 (28.2)	26 (41.3)	0.056
	No	150 (71.8)	37 (58.7)	
Sleep				
Day sleep	Likely	45 (21.5)	20 (31.7)	0.096
	Unlikely	164 (78.5)	43 (68.3)	

## Discussion

Worldwide prevalence of insulin resistance ranged from 15.5% to 46.5% [12]. This is an alarm for the future high prevalence of many chronic diseases. For this, the present study investigated the prevalence of clinical manifestations known to be associated with insulin resistance. A minority (12.9%) of the students in the sample were former or current smokers. Screening for smoking was very important as smoking is associated with insulin resistance in a dose-dependent manner. It directly increases the risk for insulin resistance, mainly via hormone activation, and may indirectly cause insulin resistance due to its effects on abdominal obesity. Nicotine may be the factor underlying these potential mechanisms [13].

In the current study, the percentage of those who have excessive day sleep was 23.9%. So going into a mini-coma and needing to take a nap after consuming too much food or a high-carb meal is a common sign of insulin resistance. This is due to both the high energy requirement of turning sugar into fat and the impact of the sugar and insulin surge on the brain's neurotransmitters [14]. Chronically high levels of insulin in the bloodstream cause the ovaries to produce more androgens, which contributes to polycystic ovarian syndrome which was 8.1% in the current study. It frequently happens that one hormone imbalance sets off an imbalance in other hormones [15].

Excessive day sleep, polycystic ovarian disease, impaired cognitive performance, and carbohydrate changes were all proven to have a significant correlation to insulin resistance [16]. The present study found that nearly one-third of female medical students had foggy brains. difficulty concentrating, struggling with planning, and solving problems, and bad memory which became worse in the past few years. These findings were supported by Willmann et al., 2020, who mentioned that insulin resistance is likely the cause of impaired cognitive performance in insulin-resistant individuals as neurodegenerative diseases may be exacerbated by deficiencies in insulin signaling in the brain [17]. Those who had excessive day sleep were 23.9% of the tested population. A study done in Jordan on medical students revealed that 50% of medical university students describe daytime sleepiness and 70% obtain inadequate sleep [18].

Those who felt hungry even after eating some sweets were around one-fourth (25.7%), but those who usually had extreme thirst or hunger were around one-third (31.3%). These findings agreed with Reche-García et al., 2022, who said that sugar cravings are a symptom of insulin resistance, which is low blood sugar brought on by high amounts of circulating insulin [19]. It creates a vicious cycle where, if one does not start eating more frequently throughout the day to minimize blood sugar falls, they will continue to have high desires, indulge, and aggravate their insulin resistance. Additionally, insulin resistance prevents glucose from the blood to enter cells, which prevents the body from converting the food you eat into energy. This lack of energy results in an increase in hunger. Polyphagia makes people feel always hungry yet eating will not let them stop feeling that way because it will only raise their blood sugar levels even more [20].

The most prevalent of the dermatological manifestations was acne, with over one-third of the study cohort having acne. Several studies support the findings of the current study. In Malaysia, the prevalence of acne among medical students was 68.1% [21], whereas another study demonstrated the prevalence of acne vulgaris to be 14.47% in female undergraduate medical students of Rawalpindi and Islamabad [22]. These differences may be attributed to genetic, environmental, or lifestyle differences.

Acne vulgaris is one of the cutaneous signs of PCOS and hyperandrogenism [23], which are seen more frequently in individuals with insulin resistance. Acne was not, however, statistically significant in its association with waist circumference as a marker of insulin resistance in the present study. This finding is not unexpected, despite the proposed relationship to insulin resistance, as acne is multifactorial and widely common among the healthy-weight population.

Among all the variables, certain other dermatological manifestations were found to be statistically significant such as acanthosis nigricans, skin tags, and alopecia areata. A study mentioned that Acanthosis nigricans neck severity grading correlates well with insulin resistance and can be used as a clinical surrogate for the assessment of the severity of insulin resistance. It is a better marker of insulin resistance than the traditionally used markers like BMI, or buffalo hump [24].

 Skin tags were significantly associated with waist circumference as a marker of insulin resistance. Although skin tags were the second most prevalent dermatological manifestation after acne, the prevalence of skin tags in the study sample was lower than expected at only 6.6% compared to the 50-60% prevalence in the general population [25]. This could be due to the lower age range of the sample size compared to the general population, seeing as skin tags increase in frequency with age. Alopecia areata followed in statistical significance with a p-value of 0.006. There has been an increase in the reporting of metabolic abnormalities in patients with alopecia areata [26], and a recent study confirmed increased serum insulin levels in patients with alopecia areata, as well as higher insulin in those with a greater number of hair loss episodes [27]. 

Manifestations such as psoriasis and hirsutism, which have well-established associations with insulin resistance [28], were not found to be statistically significantly associated with waist circumference in the present study, despite both being more prevalent in the high waist circumference category. More severe disease burden was found to be correlated to increased body mass index in hidradenitis suppurativa, and it appears that the prevalence of hidradenitis suppurativa is higher in the obese population [29]; however, the present study did not find a correlation that was statistically significant between hidradenitis suppurativa and waist circumference. PCOS, an endocrine disorder found in women of reproductive age, has a well-established link with obesity and insulin resistance. The prevalence of polycystic ovaries in the study cohort was 8.1%, which is in accordance with the prevalence of 6-10% found in the general population [30]. The study’s findings reported a p-value of 0.001, confirming the association reported in published literature.

The other parameters measured, namely cognitive changes, carbohydrate cravings, and daytime sleep, were not found to be significantly related to insulin resistance. This is despite many of these variables being reported as being higher in the high waist circumference group. This is likely due to the high prevalence of the conditions in both sub-cohorts and the role that many external factors play in influencing their prevalence. Difficulty concentrating, for example, may be due to lack of sleep or long class hours in the university, rather than insulin resistance alone. Similarly, extreme thirst is likely due to the role of the environment, since daytime temperatures are typically very high in Saudi Arabia, the location of the study.

Study limitations

Limitations of this study include that it was conducted among young female medical students, a sample that is not representative of the population due to differences in risk factors among the cohort as compared to the general Saudi population, as well as the narrow age range and lack of male participants. Study is therefore limited by selection bias. Different examiners were also used for taking anthropometric measurements, which may give rise to inter-examiner variability. Lastly, the questionnaires for dermatological changes, cognition, cravings, and sleep were based on self-reported data. Due to the subjectivity of such data, participants may under-report or over-report certain manifestations.

## Conclusions

The findings of the study suggest the association of dermatological and endocrine features such as acanthosis nigricans, skin tags, alopecia areata, and PCOS with waist circumference as an indicator of insulin resistance. The high prevalence of increased waist circumference in the study cohort is also in line with international data concerning the increasing incidence of insulin resistance and, consequently, diabetes mellitus will be seen among younger age groups. The high prevalence of insulin resistance and its dermatological signs is confirmed by the study’s findings. Patients ought to recognize the meaning of these dermatological and endocrine manifestations and make appropriate lifestyle adjustments as a result. Similarly, healthcare providers should inquire about the presence of such cutaneous signs, even in younger age demographics, and bring attention to their association with insulin resistance and diabetes. Such early identification should facilitate the prevention of the progression of diabetes mellitus and serve to hinder the escalation of this global epidemic.
